# Advances in psoriasis and gut microorganisms with co-metabolites

**DOI:** 10.3389/fmicb.2023.1192543

**Published:** 2023-11-16

**Authors:** Qiushuang Zhu, Kai Wu, Qiuhong Yang, Bo Meng, Yucun Niu, Fenglian Zhao

**Affiliations:** ^1^Department of Nutrition and Food Hygiene, Public Health College, Harbin Medical University, Harbin, China; ^2^Department of Dermatology, The 962nd Hospital of the PLA Joint Logistic Support Force, Harbin, China; ^3^Department of Chinese Medicine and Dermatology, People's Hospital of Nan Gang District, Harbin, China

**Keywords:** psoriasis, gut microorganisms, metabolites, immunity, diet

## Abstract

This review summarizes the potential role of gut microbes and their metabolites as novel mediators of psoriasis, including their composition and function in disease pathogenesis, progression, and management. Gut microbiota network analysis, colony construction, and *in vivo* large-scale interaction experiments showed that different degrees of damage and repair in psoriasis, both in animals and humans, involve cross-border homeostasis of the microbial community. Which gut microbiota interactions are present in psoriasis and how they collaborate with immune cells and influence psoriasis development via the gut-skin axis remain incompletely elucidated. In this article, we review the latest information on the unique patterns of gut microbiota and co-metabolites involved in the pathogenesis of psoriasis and attempt to explore microbial-based therapeutic targets derived from mono-and polymicrobial probiotics, fecal microbiota transplantation, pharmacomicrobiomics, and dietary interventions as diagnostic or therapeutic approaches promising to provide new options and long-term management for psoriasis.

## 1. Introduction

**Figure 1:**
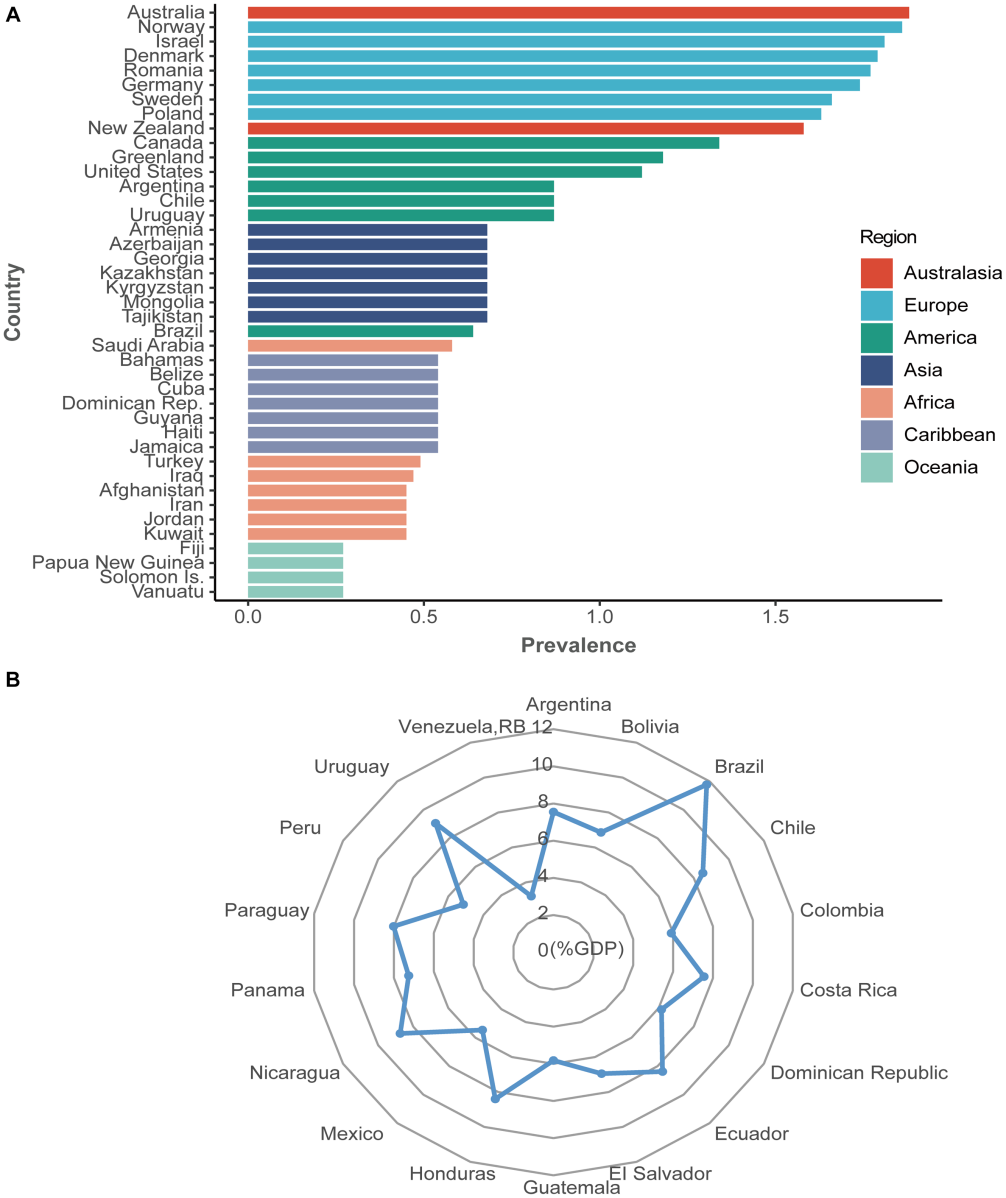
The prevalence (%) and health expenditure (%GDP) of psoriasis worldwide. **(A)** The prevalence of psoriasis from 169 countries. Seven colorful blocks represent seven continents, and each country from the belonging continent is ranked from highest to lowest prevalence, with only the top seven countries shown. The prevalence of psoriasis varied from 0.27% in Oceania to 1.88% in Australasia. **(B)** Health expenditure (%GDP) of psoriasis from 18 available countries. Health expenditure for psoriasis varied from 3.21% in Venezuela, RB, to 11.77% in Brazil. Data from the Global Psoriasis Atlas (https://www.globalpsoriasisatlas.org/). For further details about how the prevalence data is calculated, please visit https://www.bmj.com/content/369/bmj.m1590.

**Figure 2:**
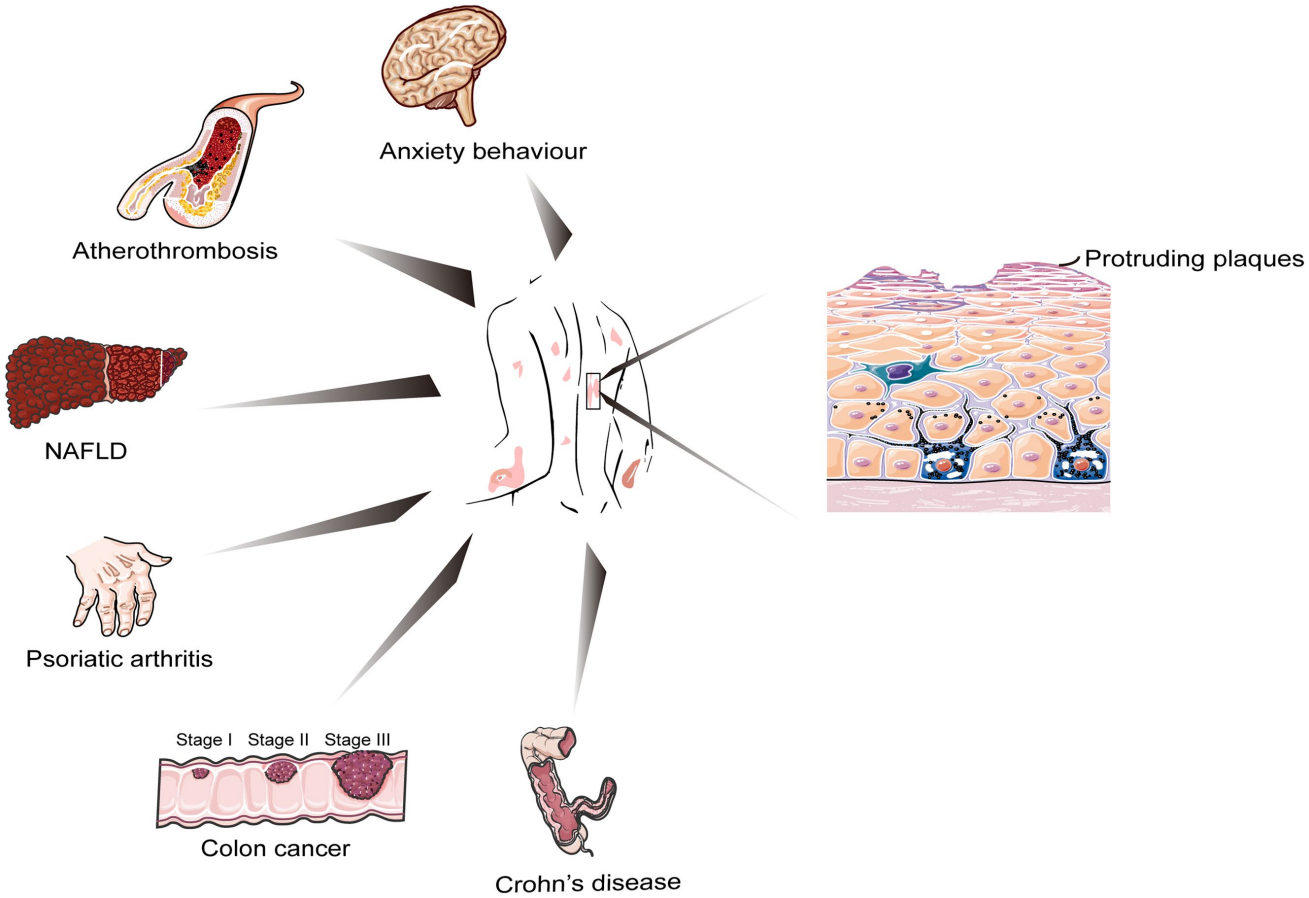
Cellular biology of psoriasis and other closely related chronic and serious health conditions.

Psoriasis, a common erythematous scaling skin disease with multiple skin manifestations and systemic involvement, can involve any skin site and occur at any age and in any geographic area, affecting more than 60 million adults and children worldwide (Griffiths et al., 2021; Figure 1; Supplementary Tables S1, S2). Based on different clinical manifestations of psoriasis, it is usually classified into five types: plaque-dominated psoriasis vulgaris, punctate (droplet) or hemorrhagic psoriasis, pustular psoriasis (represented by sterile pustules), arthritic psoriasis (with arthritis as the main manifestation), and erythrodermic psoriasis with systemic involvement, of which psoriasis vulgaris is the most common type, accounting for approximately 90% of cases (Boehncke and Schön, 2015). Immunological and genetic studies confirmed IL-17 and IL-23 as key drivers in psoriasis pathogenesis (Ghoreschi et al., 2021). However, psoriasis is currently incurable due to its lingering and recurrent nature. A plethora of studies found that psoriasis is no longer considered a disease that affects only the skin but is seen as a systemic inflammatory disorder (Gulliver, 2008; Reich, 2012; Elnabawi et al., 2019; Gelfand and Wang, 2023), which is associated with multiple comorbidities, including colorectal cancer, metabolic syndrome, obesity, nonalcoholic fatty liver disease, and cardiovascular disease (Griffiths and Barker, 2007; Figure 2). Fu et al. (2018) published a large meta-analysis of data from nearly 7.8 million people, which showed that psoriasis was significantly associated with both Crohn’s disease and ulcerative colitis. Two years later, another meta-analysis on the results of eight cohort studies (10,544,609 subjects in total) found a significantly increased risk of colorectal cancer in women with psoriasis (but not men), suggesting that patients with psoriasis exhibiting gastrointestinal symptoms should undergo colonoscopy (Fu et al., 2021).

Microbial infections are considered an important etiology of psoriasis, especially upper respiratory tract infections with streptococci, which are strongly associated with the development of psoriasis vulgaris (De Jesús-Gil et al., 2020). However, as the understanding of psoriasis improved, both *in vivo* experiments and clinical trials demonstrated that certain common genetic and environmental factors and immune pathways might be present in psoriasis and inflammatory diseases due to intestinal dysbiosis (Schreiber et al., 2019; Okada et al., 2020; Paine et al., 2023). Indeed, the gut microbiota is a community of trillions of symbiotic organisms that work together in metabolically active endocrine-like organs now known to contribute to host physiology through the digestion of many nutrients, vitamin synthesis, and production of bioactive metabolites (Benson et al., 2023). Therefore, the association of human gut microbiota and commensal metabolites with psoriasis is receiving attention. Additionally, studies showed that various microbiota metabolites, such as short-chain fatty acids (SCFAs), tryptophan metabolites, and amine derivatives, including trimethylamine N-oxide (TMAO), play an important regulatory role in autoimmune diseases (Stec et al., 2023). The composition of gut microbes influences nutrition, inflammation, natural immune function, and, to some extent, people’s skin condition.

Given that intestinal dysbiosis is involved in the physiopathology of inflammatory and immune diseases, the correction of intestinal dysbiosis and the maintenance of intestinal microecological balance are new targets for the prevention and treatment of psoriasis. Therefore, in this paper, we explored microbial therapy for psoriasis, including mono-and polymicrobial probiotics, fecal microbiota transplant (FMT), pharmacomicrobiomics, and dietary interventions. More importantly, we not only summarized the effective cure rate, prognosis with single or combined probiotics, and molecular mechanisms by which the gut microbiota might be engaged in drug metabolism in the treatment of psoriasis but also evaluated the effects of probiotics interacting with exogenous dietary factors on psoriasis, providing evidence for microbial immunotherapy as well as dietary interventions.

## 2. Gut microbiome and psoriasis

In the human body, the skin and the intestine are the two organs with the most abundant microbiota, and the gut microbiota is involved in the occurrence and development of human systemic diseases in many ways. Many reports confirmed the close association between gut microbiota and diseases such as cancer (Cheng et al., 2020), type 2 diabetes (Qi et al., 2022), mental illness (Sampson et al., 2016), obesity (Liu et al., 2017), and autoimmune diseases, including lupus erythematosus and rheumatoid arthritis (RA; Miyauchi et al., 2023). The gut microbes have a crucial role in maintaining the integrity of the intestinal mucosal barrier, immune homeostasis, and the dynamic balance of energy and helping the host absorb vitamins; hence, the mesenteric system is also considered the largest immune organ in the body (Jacobs and Braun, 2014).

A strong bidirectional link between the gut and skin has been demonstrated, with many studies linking gastrointestinal health to dynamic homeostasis and heterogeneity of the skin. Patients with psoriasis have a reduced diversity of intestinal microbiota and possible dysbiosis compared to healthy controls, as evidenced by the changes in the microbiota at different levels of categorization, as detailed in Table 1 (Scher et al., 2015; Eppinga et al., 2016; Codoñer et al., 2018; Tan et al., 2018; Hidalgo-Cantabrana et al., 2019; Huang et al., 2019; Shapiro et al., 2019; Dei-Cas et al., 2020; Xiao et al., 2021; Zhang et al., 2021). Hidalgo et al. sequenced and analyzed 16S rRNA in the fecal samples of 19 patients with psoriasis and 20 healthy individuals from the same region and found a low diversity and dysbiosis of gut microbiota in patients with psoriasis (Hidalgo-Cantabrana et al., 2019). The structural changes in the microbiota of psoriasis patients in this study were specific and elucidated the mechanisms of gut microbiota-host interactions in psoriasis. Kiyohara et al. induced psoriasis-like dermatitis using TLR7 agonist in the skin of mice, affecting intestinal immune cell and microbiota composition, which, in turn, led to an exacerbation of DSS-induced colitis (Kiyohara et al., 2018). Additionally, a study found that type IIA secreted phospholipase A2 (sPLA2-IIA) modulates gut microbiota in mice by degrading bacterial membranes, altering the expression of intestinal immune and metabolism-related genes, and regulating the levels of multiple blood metabolites and fecal bacterial lipids to suppress bacterial infections and alleviate DMBA/TPA-induced skin cancer while worsening imiquimod (IMQ)-induced psoriasis (Miki et al., 2022). These findings suggest that in the gut-skin axis, skin inflammation can affect gut health, and gut microbiota can affect skin health, shedding new light on the relevance of psoriasis to inflammatory bowel disease. Similar results were observed by Schneeweiss et al., who analyzed more than 240,000 patients with chronic inflammatory skin disease and nearly 2.4 million controls and found that the risk for ulcerative colitis and Crohn’s disease was significantly higher in patients with septic sweat glands and risk for Crohn’s disease was significantly higher in patients with psoriasis (Schneeweiss et al., 2022).

**Table 1:** Intestinal dysbiosis in psoriasis.

Study	Subjects	Sample	Gut Microbiota Analysis Technique	Gut microbiota alterations
Hidalgo-Cantabrana et al. (2019)	19 psoriasis patients; 20 healthy individuals	Fecal samples	16S rRNA	**Phylum**: *Actinobacteria, Firmicutes, Bacteroidetes, Proteobacteria* **Genus**: *Blautia, Bifidobacterium, Collinsella, Ruminococcus, Subdoligranulum, Slackia; Bacteroides, Parabacteroides, Barnesiella, Alistipes, Paraprevotella, Faecalibacterium*
Zhang et al. (2021)	30 psoriasis patients; 30 healthy controls	Fecal samples	16S rRNA	**Family**: *Veillonellaceae, Ruminococcaceae; Lachnospiraceae* **Genus**: *Faecalibacterium, Megamonas*
Scher et al. (2015)	15 patients with psoriasis of the skin;17 healthy subjects	Fecal samples	16S rRNA	**Genus**: *Parabacteroides, Coprobacillus*
Huang et al. (2019)	35 psoriasis patients;27 healthy controls	Fecal samples	16S rRNA	**Phylum**: *Bacteroidetes; Firmicutes* **Genus**: *Bacillus, Bacteroides, Sutterella, Lactococcus, Lachnospiraceae_UCG004, Lachnospira, Mitochondria_norank, Cyanobacteria_norank, Parabacteroides; Thermus, Streptococcus, Rothia, Granulicatella, Gordonibacter, Allobaculum, and Carnobacterium*
Tan et al. (2018)	14 patients with vulgaris psoriasis; 14 healthy controls	Fecal samples	16S rRNA	**Phylum**: *Verrucomicrobia, Tenericutes* **Genus**: *Enterococcus, Bacteroides; Akkermansia*
Xiao et al. (2021)	30 psoriatic patients;15 healthy subjects	Fecal samples	Metagenomic sequencing	**Phylum**: *Actinobacteria, Firmicutes, Verrucomicrobia; Bacteroidetes, Proteobacteria* **Genus**: *Faecalibacterium, Bacteroides, Bifidobacterium, Megamonas,Roseburia*; *Prevotella, Alistipes, Eubacterium*
Eppinga et al. (2016)	29 psoriasis patients; 33 healthy individuals	Fecal samples	quantitative polymerase chain reaction	**Species**: *Escherichia coli; Faecalibacterium prausnitzii*
Codoñer et al. (2018)	52 plaque psoriasis;300 healthy individuals extracted from the human microbiome project (http://hmpdacc.org/)	Fecal samples	16S rRNA	**Genus**: *Faecalibacterium, Akkermansia* spp.*; Bacteroides*
Shapiro et al. (2019)	24 psoriasis patients; 22 healthy individuals	Fecal samples	16S rRNA	**Phylum**: *Firmicutes, Actinobacteria* **Species**: *Ruminoccocus gnavus, Dorea formicigenerans, Collinsella aerofaciens; Prevotella copri, Parabacteroides distasonis*
Dei-Cas et al. (2020)	55 psoriasis patients; 27 healthy individuals	Fecal samples	16S rRNA	**Phylum**: *Firmicutes; Bacteroidetes* **Genus**: *Faecalibacterium, Blautia; Bacteroides, Paraprevotella*

Red font – increased level; blue font – decreased level.

Exploring the changes and functions of the gut microbiota in psoriasis can help provide new targets for the diagnosis and treatment of psoriasis. Studies showed that helper T cells (Th17) are important mediators of intestinal epithelial barrier integrity and can drive both intestinal inflammation and extraintestinal autoimmune disease progression through the microbiota, acting as a bridge between host microbiota and immune-mediated inflammatory diseases; thus, the heterogeneity and plasticity of Th17 are expected to be a breakthrough in the mechanisms of psoriasis development and refinement of “litmus test” therapeutic strategies (Bellone et al., 2020; Schnell et al., 2023). For example, different species of the same genus differ in their regulation of Th17 in different environments. *P. histicola* alleviates RA (Marietta et al., 2016), and *P. copri* exacerbates arthritis (Scher et al., 2013). Ito et al. found that *Staphylococcus cohnii*, a commensal bacterium present in mouse and human skin, could suppress skin inflammation in several mouse models of dermatitis by inducing the expression of anti-inflammatory genes and glucocorticoid-related pathway genes (Ito et al., 2021). Oral administration of *Lactobacillus pentosus* GMNL-77 significantly decreased the interleukin (IL)-23/IL-17A axis–associated cytokines and erythematous scaling lesions in the skin of IMQ-treated mice, suggesting that artificial alteration of the gut microbiota might be relevant for reducing the systemic inflammatory response in the skin of psoriasis patients (Chen et al., 2017).

Although biologics have achieved high skin lesion clearance in psoriasis, the current process of treating psoriasis with biologics has been a source of secondary failure problems and safety issues have been a major concern for clinicians. Clinical evidence showed that IL-23 inhibitors reachive remission in patients who do not respond well to IL-17 monoclonal antibodies (Sofen et al., 2014; Reich et al., 2019). Significant differences in the relative abundance of bacteria taxa between responders and non-responders suggested that IL-23 and IL-17 inhibitors may functionally interact with gut microbiota to reduce cutaneous inflammation (Huang et al., 2023). However, the specific mechanisms and applications of the microbiome in psoriasis treatments require more attention.

## 3. Fungi and psoriasis

In addition to bacteria that can influence various physiopathological processes from immune development to phenotype, mounting studies demonstrated the involvement of intestinal fungi in the regulation of immune homeostasis (Doron et al., 2021; Li B. Z. et al., 2022). Fungi are non-negligible members of the gut microbiota. Composition changes of intestinal fungi were found in various diseases, such as systemic lupus erythematosus, RA, and colitis (Sokol et al., 2017; Li H. V. et al., 2022). However, the effects of fungi on host health and their mechanisms of action are still poorly understood. Adult intestinal fungi are mainly composed of 10 genera in the phylum Cysticercus (70%) and Streptomyces (30%) and are influenced by environmental and dietary factors (Richard and Sokol, 2019). There are two main types of fungi found in the human body: environmental fungi, such as yeasts and molds, which are usually harmless to healthy people, and commensal fungi. The latter live in the human skin or body and can improve intestinal health. Conversely, changes in the composition of intestinal fungal (called “fungal dysbiosis”) can lead to colon cancer, alcoholic liver disease, and allergic respiratory disease (Wheeler et al., 2016; Lang et al., 2020; Papon et al., 2021). Therefore, there is a possible role of a balanced intestinal fungal community in maintaining the dynamic balance of host immunity and human health.

Although gut bacteria and development of psoriasis have bidirectional regulatory mechanisms, core questions regarding the involvement of fungal biota are only beginning to be investigated. A case–control study found that early skin infections, including cutaneous viral, bacterial, and fungal infections, and microbial ecological dysbiosis were significantly associated with psoriasis in children (Chen Y. J. et al., 2021). Th cells play different roles in fungal infection and colonization (Speakman et al., 2020). Hurabielle et al. found that colonization by skin commensal fungi, such as *Candida albicans*, worsened psoriasis-like skin inflammation by enhancing the response of Th17 cells, neutrophils, and Langerhans cells and inducing changes in the skin transcriptome of mice similar to those in lesional skin of psoriasis patients (Hurabielle et al., 2020). Besides, another study showed the presence of a specific fungal community in the intestinal mucosa of humans and mice and that mucosa-associated fungi protect the intestine from damage and infection through the induction of IL-22 by Th17 cells (Leonardi et al., 2022). In addition to this, *Pseudomonas tropicalis* can promote colorectal tumorigenesis through various mechanisms, such as modulation of host immunity, induction of inflammatory vesicle production, myeloid-derived suppressor cell differentiation, and IL-22 secretion by ILC3 cells (Wang et al., 2018). Due to their large size, fungi can also compete with bacteria in the intestine and inhibit the proliferation of probiotics, thus, promoting tumorigenesis and progression (Narunsky-Haziza et al., 2022).

The mechanisms of intestinal fungi involvement in psoriasis have rarely been reported, but these aforementioned findings revealed the strain specificity of host-fungal interactions and highlighted new diagnostic and therapeutic targets for diseases of inflammatory origin. It is thus clear that deciphering the numerous interactions between bacteria and fungi in the gut and other ecological settings is one of the most explored areas of research in the gut-skin axis, and a deeper understanding of the mechanisms of immune regulation mediated by fungal-bacterial and fungal-fungal interactions will help elucidate their role in the development of psoriasis.

## 4. Commensal metabolites and psoriasis

One of the main mechanisms by which gut microbes play a role in autoimmunity and stimulation of immune responses is the alteration of microbial metabolites with immunomodulatory functions (Arpaia et al., 2013). Thus far, only a few metabolites have been identified and extensively studied, such as SCFAs, tryptophan, and secondary bile acids, which play specific roles at the cellular and even systemic level by interacting with different receptors on immune and skin cells (Campbell et al., 2020; Choi et al., 2020; Schwarz et al., 2021). Understanding how microbial-host symbiotic metabolites affect the skin and immune cells may be a milestone in deciphering the mechanisms of the gut-skin axis.

Gut microbiota has an important role in the maintenance of systemic immune homeostasis and can affect skin health in many ways, including immune regulation and strain/metabolite transfer. A recent study has found that sodium butyrate treatment alleviated skin inflammation, decreased IL-17 expression, and increased IL-10 and Foxp3 expression in a mouse model of psoriasis. In Treg isolated from the blood of patients with psoriasis, sodium butyrate restored the acetylation level of its inhibitory agent H3 histone. In the lesional skin of patients with psoriasis, sodium butyrate restored Treg numbers and dysregulated levels of certain cytokines, such as IL-17 (Schwarz et al., 2021).

Notably, patients with psoriasis often have impaired intestinal barrier integrity and altered gut microbiota and are seven times more likely to develop inflammatory bowel disease (IBD) than the general population, although the responsible mechanisms are unclear. A recent study has found that psoriasis disrupts gut microbiota, causing the production of succinate and pro-inflammatory ligands by intestinal microorganisms, which induce the proliferation and activation of colonic CX3CR1hi macrophages, ultimately exacerbating colitis (Pinget et al., 2022). Additionally, there is an important link between the metabolism and immune responses of the body. A study showed that a high-fat diet causes changes in the metabolic state of the body, which in turn, alter the activation of Toll-like receptor (TLR)-dependent dendritic cells, increases IL-23, and ultimately exacerbates the inflammatory symptoms of psoriatic skin (Mogilenko et al., 2019). Therefore, intestinal microorganisms and their metabolites might break through the damaged intestinal barrier to enter the body circulation and, thus, directly or indirectly regulate distant organs, including the skin and joints, and play an important role in the process of establishing, dysregulating, and re-establishing homeostasis in the gut-skin axis.

Trimethylamine (TMA) is generated from dietary carnitine and choline in fish, eggs, and beef products by intestinal microbiota choline TMA lyase. Then, TMA enters the liver via the portal circulation and is oxidized by the hepatic enzyme flavin-containing monooxygenase 3 to form TMAO (Cho and Caudill, 2017). Hazen’s team initially discovered that increased TMAO levels are associated with an increased risk of incident major adverse cardiovascular events (Wang et al., 2011; Tang et al., 2013). A recent prospective cohort study of 6,785 participants followed for approximately 17 years confirmed that higher plasma TMAO levels were associated with a higher risk of all-cause mortality, risk of cardiovascular disease mortality, and risk of renal failure mortality and were not significantly associated with risk of cancer and dementia mortality (Wang et al., 2023). Researchers at the University of Cincinnati, USA, who enrolled 2,129 individuals from 2 independent cohorts, confirmed that plasma TMAO levels are positively correlated with the risk of abdominal aortic aneurysm (AAA) onset and progression and elucidated the mechanism by which gut microbiota-derived TMAO enhances endoplasmic reticulum stress and apoptosis in smooth muscle cells of the aortic wall, leading to the development of AAA in mouse experiments (Benson et al., 2023). The American College of Cardiology/American Heart Association identified psoriasis as an independent risk factor for atherosclerosis, myocardial infarction, and stroke (Grundy et al., 2019). Whether circulating TMAO, also an independent risk factor for cardiovascular disease, is involved in the pathogenesis of psoriasis remains a mystery. Several clinical trials demonstrated that TMAO could be used as an indicator of psoriasis severity by measuring TMAO in the serum of psoriasis patients by high-performance liquid chromatography-mass spectrometry (Coras et al., 2019; Sikora et al., 2021; Sun et al., 2022). In a mouse model of systemic lupus erythematosus, the intestinal microbial metabolite, TMAO, contributes to TLR7-induced autoimmune and vascular dysfunction through the activation of pro-inflammatory Th17 lymphocytes and an increase in B cell differentiation (González-Correa et al., 2021). It remains to be determined whether TMAO plays a marker or mediator role in the etiology of psoriasis, whether its high concentration can activate the immune system, and whether it can coexist with factors of homeostasis within the circulatory system.

New biologic treatments have recently been added to psoriasis treatment options (e.g., bimekizumab, secukinumab, and ixekizumab; Burkett and Kuchroo, 2016). Although effective, clinical randomized controlled trials demonstrated that adverse events were reported in 86.1% of patients receiving bimekizumab and 81.4% of patients receiving secukinumab between 48 weeks, with bimekizumab leading to a higher incidence of ulcerative colitis, oral candidiasis, and suicide risk (Reich et al., 2021). Therefore, the highly heterogeneous disease profile of various biologics still requires more exploration in terms of efficacy and safety. As increasing evidence supports the importance of the microbiome for our health, the desire to promote a healthy microbiome becomes stronger. In addition to TMAO, there are many favorable gut microbiota-derived metabolites such as butyrate (Wen et al., 2021), propionic acid (Duscha et al., 2020), and tryptanthrin (Shankar et al., 2020). These metabolites, as postbiotics for the therapeutic purpose of autoimmune diseases, are mainly aimed at correcting dysbiosis and the imbalance between resident microorganisms and the immune system that contribute to health risks. Postbiotics are the latest trend in gut health, promising to improve our skin (Rawal and Ali, 2023), enhance our physique (Akatsu, 2021), and even reverse the signs of aging (Iglesia et al., 2022).

## 5. Drug-microorganism interference

As pharmacomicrobiomic studies progressed, it was discovered that gut microbiota could be used as a biomarker for predicting therapeutic response. Additionally, modulating microbiota could increase the bioavailability and efficacy of drugs, and inhibiting the enzymatic activity of specific bacteria could prevent them from metabolizing drugs into toxic products. The heterogeneity of the microbiome among individuals could determine the clinical efficacy of certain drugs or reduce the occurrence of adverse events, which is well used in RA, psoriatic arthritis, and ankylosing spondylitis (Scher et al., 2020).

Methotrexate, used to treat colon cancer and psoriasis, is also used in the treatment of RA (Marsh et al., 1991; van Huizen et al., 2022; Zhang et al., 2022), but about 50% of patients with RA do not respond adequately to methotrexate therapy. A clinical study (Artacho et al., 2021) comparing pretreatment differences in gut microbiota between RA patients who responded and those who did not respond to methotrexate therapy found that a microbiota-based model could more accurately predict patient response to methotrexate therapy. Additionally, *in vitro* co-culture of gut microbiota with methotrexate suggests that the metabolism or clearance of methotrexate by gut microbiota might inhibit the therapeutic effect of methotrexate. Another study (Ventin-Holmberg et al., 2021) comparing the differences in fecal microbiota (both bacterial and fungal) before and after infliximab treatment found that non-responders had lower abundance of short-chain fatty acid-producing bacteria (especially *Clostridium*) and higher abundance of pro-inflammatory bacteria and fungi (such as *Candida* spp.) compared to responders, and that response to infliximab treatment was more accurately predicted based on bacterial taxa.

**Figure 3:**
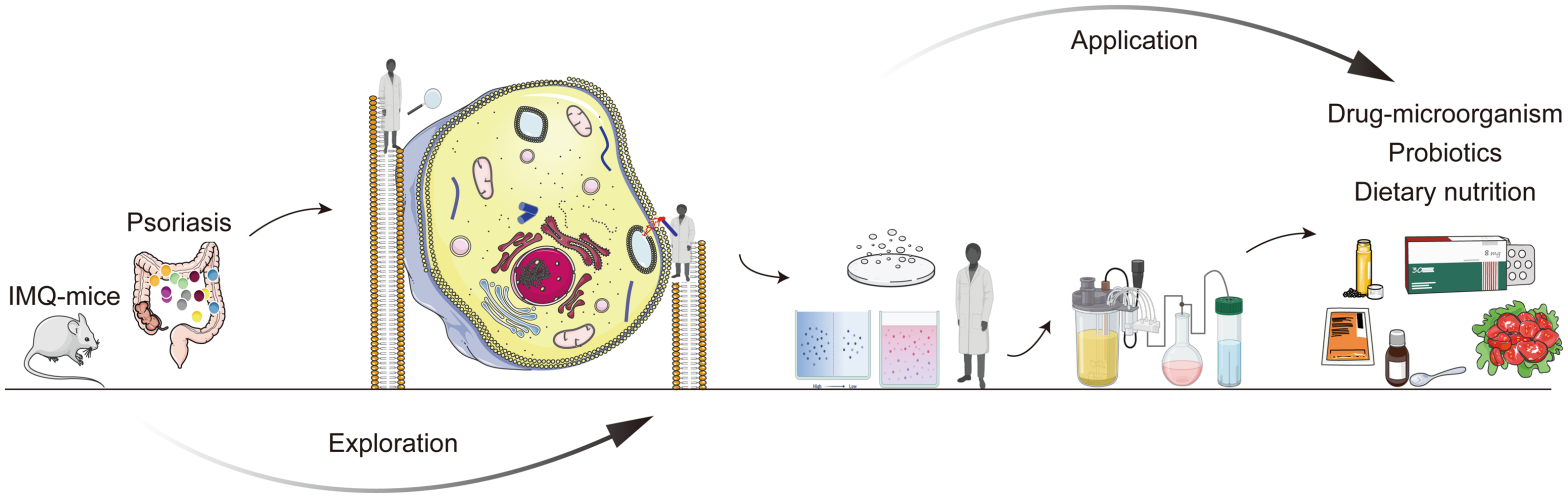
Schematic representation of “ignorance to knowledge” of gut microbiota and psoriasis.

Zimmermann et al. (2019) systematically analyzed the metabolism of 271 orally administered drugs by 76 species/strains of human intestinal bacteria, identified bacterial genes and their products involved in drug metabolism, and validated some of the findings in mouse models and human gut microbiota cultures, deepening the understanding of the molecular mechanisms involved in drug metabolism by gut microbiota and providing insights into individualized drug interventions targeting microbiota. Furthermore, since psoriasis and IBD are highly heterogeneous diseases, more precise and in-depth phenotyping is needed to identify specific subgroups and their molecular signatures as human microbiome research advances. Scientists are beginning to identify core and variant microbiomes (enteric, vagal, and metabolic; Rizkallah et al., 2010), how different functional groups can be precisely combined with drugs, and how interventions (e.g., microbiome editing) and clinical practice (e.g., microbiome testing) can be administered to treat autoimmune diseases. Although a huge challenge, there is no doubt that the translation of pharmacomicrobiomics into routine healthcare applications is just around the corner (Figure 3).

## 6. Microbiotherapy and psoriasis

A retrospective analysis showed that 83.7% of psoriasis patients had 100% improvement in PASI scores after 24 weeks of treatment with *Streptococcus salivarius* K-12 and that efficacy continued to improve with longer treatment duration (Zangrilli et al., 2022). Besides, a randomized controlled trial found that continuous oral administration of *Bifidobacteria infantis* (*B. infantis*) 35,624 significantly improved the progression of psoriasis and reduced the expression of C-reactive protein and tumor necrosis factor (TNF)-α, showing that the immunomodulatory effects of the microbiota in humans are not limited to the mucosal immune system but extend to the systemic immune system (Groeger et al., 2013). In addition to this, there are also animal studies that demonstrate that individual probiotics can improve the symptoms of psoriasis (Chen et al., 2017, 2023). GMNL-77 decreased skin erythema and scaling, inhibited hyperplastic suprabasal keratinocytes, suppressed mRNA expression of pro-inflammatory cytokines, including TNF-a, IL-6, and the IL-23/IL-17A axis–associated cytokines (IL-23, IL-17A/F, and IL-22), in skin lesions, decreased the spleen weight, and also reduced the numbers of IL-17/IL-22-producing CD4^+^ T cells in the spleen (Chen et al., 2017). Furthermore, a probiotic mixture can also improve the symptoms of psoriasis, but the mechanism behind it is complex and variable (Navarro-López et al., 2019; Chen H. L. et al., 2021; Choy et al., 2023).

Recent study has found that the transfer of intestinal microbiota from mice with severe psoriasis-like skin phenotype exacerbated psoriasiform skin inflammation in mice with mild symptoms, including increasing the infiltration and differentiation of Th17 and the abundance of Prevotella while decreasing that of *Parabacteroides distasonis* in the colon. These alterations affected fatty acid metabolism, increasing the abundance of oleic and stearic acids. In turn, administration of oleic and stearic acids exacerbated psoriasis-like symptoms and increased Th17 and monocyte-derived dendritic cell infiltration in the skin lesion areas *in vivo*, as well as increased the secretion of IL-23 by stimulating dendritic cells (DCs) *in vitro*. Therefore, the influence of exogenous dietary factors has to be considered while using probiotics (Zhao et al., 2023). Additionally, do probiotics have side effects, can probiotics colonize the intestine, and what is the quality of survival? How long does it take for probiotics to work? Can it interact with other microorganisms and their metabolites in different intestinal segments? The mechanism of probiotics is still to be fully analyzed (Suez et al., 2019). After the mechanism of single probiotics is clearly explained, the application of probiotics in combination and the corresponding standards and methods still need to be explored. We have different diets and environments, which might cause differences in gut microbiota. Hence, when to use probiotics and their precise application according to different individuals are still to be further developed.

Single strains have made many advances in the treatment of diseases with localized lesions, such as plaque psoriasis. Meanwhile, multistrain therapy, such as FMT, is considered a method to correct gut microbiota dysbiosis and re-establish intestinal microecological balance. Such therapy has been used in recent years to treat psoriasis (Yin et al., 2019), systemic lupus erythematosus (Huang C. et al., 2022), irritable bowel syndrome (El-Salhy et al., 2020), and Parkinson’s disease (Zhao et al., 2021), and even influences the response to cancer immunotherapy (O'Leary, 2021). Furthermore, Chen et al. found that the imiquimod-induced psoriasis in mice with healthy donors stool exhibited effective antipsoriatic skin inflammation and even two individual humanized mice almost completely abrogated skin lesion progression (Chen Y. J. et al., 2021). However, the effectiveness of FMT treatment varies depending on the different diseases, the form and number of grafts, the route of administration, and the donor used (Green et al., 2020; Ianiro et al., 2022). An exploratory randomized placebo-controlled trial of 31 patients with active peripheral psoriatic arthritis (PsA) underwent either fecal transplantation or sham surgery. Twenty-six weeks of clinical evaluation showed that fecal transplantation had a good safety profile but had a higher rate of treatment failure than sham surgery and was inferior or non-inferior to sham surgery on secondary measures, such as HAQ-DI scores and ACR20 (Kragsnaes et al., 2021). Moreover, the response rate at 6 months after FMT in patients with irritable bowel syndrome was only 27.5% (Huang H. L. et al., 2022). Although FMT is known to multiply recurrent Clostridioides difficile (mrCDI), greater interest has been drawn to whether the altered microbiota of the recipient affects their risk of other diseases (e.g., psoriasis). Based on a comparison of data from 1,165 CDI patients treated with FMT and 3,692 mrCDI patients who did not undergo FMT, it was found that FMT had no significant association with diabetes, hypertension, or psoriasis, but it increased the risk of myocardial infarction by 68% (Dawwas et al., 2022).

## 7. Dietary nutrition and psoriasis

Drugs and probiotics alone are not effective in all cases. It has been confirmed that diet has a significant impact on gut microbiota diversity in skin disease patients and normal people (Simpson et al., 2022; Barati et al., 2023). The most recent research found that high-fat diets rather than carbohydrates or proteins exacerbate psoriatic skin inflammation by altering the mucus barrier and gut microbiota, resulting in an enhanced systemic IL-17 response, which exacerbates psoriasis (Sonomoto et al., 2023). However, the fact that a high-fat diet exacerbates tissue inflammatory diseases such as psoriasis may also stem from the molecular mechanisms by which dietary components and tissue lymphocyte responses interact. It has been shown that IL-17-producing γδ T cells in the skin need to sense cholesterol metabolites (hydroxysterols) via GPR183 to maintain their thymic development and skin homeostasis, and that dietary cholesterol promotes the activation of these cells and worsens skin inflammation in mice (Frascoli et al., 2023). A recent study (Shi et al., 2020) has found that mice fed a Western-style diet for a short period of time exhibited IL-17A-mediated skin inflammation before significant weight gain occurred. Mechanistically, the Western diet induces psoriasis-like dermatitis by disrupting the homeostasis of IL-23 and bile acid signaling pathways, promoting γδT cell infiltration at the skin and enhancing their ability to produce IL-17A. Furthermore, a high-fat diet increases free fatty acids, which inhibit TLR-activated hexokinase activity and interfere with the tricarboxylic acid cycle, thereby enhancing the production of mitochondrial reactive oxygen species (mtROS), increasing the unfolded protein response, altering cellular transcription, and increasing IL-23, ultimately exacerbating the inflammatory symptoms of psoriatic skin (Mogilenko et al., 2019). In spite of this, it is still unclear what the long-term health effects of dietary fat will be. Shi et al. demonstrated that an isocaloric moderately high-fat diet extends lifespan in male rats and Drosophila (Shi et al., 2021). As compared to those on an high-carbohydrate, low-fat (HCLF) diet, T2DM patients on a 6-month, calorie-unrestricted, low-carbohydrate, high-fat (LCHF) diet had greater clinically meaningful improvements in their glycemic control and weight. In order to truly benefit your health, you need to adhere to dietary changes over time, as the changes were not sustained 3 months after intervention (Hansen et al., 2023). In addition, high-protein diets may improve glucose homeostasis *in vivo* by promoting glucose tolerance via upper small intestinal peptide transporter 1 and inhibiting gluconeogenesis (Dranse et al., 2018). However, intake of different sources of dietary protein has different associations with long-term cause-specific mortality and chronic disease prevalence. Higher intake of plant proteins is associated with significantly lower cardiovascular disease risk and mortality (Huang et al., 2020). Replacement of red meat with high-quality plant protein foods (legumes and nuts, etc.) improves lipid and lipoprotein indices and inflammatory burden (Guasch-Ferré et al., 2019; Hruby and Jacques, 2019).

Psoriasis, a chronic inflammatory skin disease, has significant associated morbidity and impact on quality of life. In addition to phototherapy, biologic agents, and microbial therapies, it is important to determine whether dietary interventions can help reduce disease severity in psoriasis. Although both short-and long-term very low–calorie ketogenic diets (VLCKDs) have certain side effects, such diet significantly reduces inflammation and is an effective means of relieving symptoms in obese psoriasis patients, possibly related to the microbiota-gut-skin axis (Barrea et al., 2022). A French questionnaire cohort study of 35,000 people, using the MEDI-LITE score to assess adherence to the Mediterranean diet, showed a significant negative association between the MEDI-LITE score and severe psoriasis, suggesting that the Mediterranean diet might slow psoriasis progression (Phan et al., 2018). Moreover, the Medical Board of the National Psoriasis Foundation offers scientifically sound and detailed recommendations for the diet of adults with psoriasis or PsA, including gluten-free diet in psoriasis, dietary weight reduction, and dietary supplements (e.g., fish oil, vitamin D, selenium, and micronutrient supplementation; Ford et al., 2018).

## 8. Conclusion and future perspectives

In this article, we provided current evidence on the role of the gut microbiome and metabolites in psoriasis and discussed their potential implications for diagnosis and treatment. Significant progress has been made in characterizing the composition of gut microbes and their relevance to inflammatory diseases of the skin, as well as in resolving whether microbes interact with host cells through various small molecules and signaling peptides. Furthermore, a number of emerging microbial interventions/therapeutic strategies and protocols for their clinical application emerged. However, there are still many challenges in facing the important scientific issue of “relapse after drug withdrawal” in psoriasis. Therefore, further exploring the pathogenesis of psoriasis and screening for new targets, new candidate commensal bacteria, and their metabolite molecules that are safe and effective in prolonging the time to relapse are greatly significant.

## Author contributions

QZ and KW collected the data research from PubMed and proposed the structure of the manuscript. QZ wrote the first manuscript draft. QY and BM contributed to funding acquisition. FZ and YN had primary responsibility for the final content. All authors contributed to the article and approved the submitted version.
